# Relationship between asymptomatic rotavirus infection and jaundice in neonates: a retrospective study

**DOI:** 10.1186/s12887-018-1352-z

**Published:** 2018-11-30

**Authors:** Nu Ri Hwang, Jin Kyu Kim

**Affiliations:** 1 Department of Pediatrics, Chonbuk National University Medical School, 20, Geonjiro Deokjin-gu, Jeonju-si, Jeollabuk-do 54907 South Korea; 2 Research Institute of Clinical Medicine of Chonbuk National University–Biomedical Research Institute of Chonbuk National University Hospital, Jeonju, Korea

**Keywords:** Jaundice, Neonatal intensive care unit, Rotavirus infection

## Abstract

**Background:**

Rotavirus (RV) infection in neonates can be mild or even asymptomatic. In RV infection, jaundice is often reported, but the relationship between jaundice and RV infection has not been studied. This study aimed to determine the importance of asymptomatic RV screening in neonates with jaundice.

**Methods:**

Neonates from the neonatal intensive care unit (NICU) of Chonbuk National University Hospital, those transferred from local obstetrics and gynecology hospitals and outpatient clinics were selected from 2014 to 2017. The study included only infants aged between 3 and 28 days. Jaundice was defined according to gestational age and birth age, in accordance with the American Academy of Pediatrics guidelines criteria. RV infection was confirmed by a stool test, and RV screening and laboratory tests were performed at admission.

**Results:**

Among 596 patients, 166 patients had jaundice. RV infection was observed in 70 (42%) jaundice patients. There were 36 (22%) jaundice patients with asymptomatic RV infection. Patients with onset of jaundice 3–7 days after birth had a high incidence of RV infection. When the RV test was positive, the risk of jaundice was significantly high [odds ratio (OR) 1.89; 95% confidence interval (CI), 1.20–2.98; *p* = 0.006].

**Conclusions:**

Infants with the onset of jaundice > 3 days after birth were likely to have RV infection. Therefore, we suggest that screening tests for RV infection be included as part of the evaluation of jaundiced infants presenting to NICU.

**Electronic supplementary material:**

The online version of this article (10.1186/s12887-018-1352-z) contains supplementary material, which is available to authorized users.

## Background

Rotavirus (RV) is the most common cause of viral gastroenteritis in children, and 43–78% of asymptomatic neonatal infections have been described [1]. Despite being predominantly asymptomatic, neonatal RV infection has received considerable attention, because some studies have reported that natural infection in the neonatal period has been shown to confer protection against subsequent severe diseases [2]. Although it is known that jaundice occurs in premature infants due to RV infection, studies on whether RV infection causes jaundice in neonates are lacking [3, 4].

Jaundice itself is not a disease but rather a symptom or sign of a disease [5]. Many conditions may clinically present as prolonged neonatal jaundice, including neonatal hepatitis and extrahepatic biliary atresia. These conditions have numerous infectious, metabolic, and genetic causes [6]. Multiple studies have described patients with proven bacterial infections who developed jaundice during the course of their illness [7, 8]. In previous studies, urinary tract infection was observed in 12.5% of asymptomatic jaundiced neonates, with the onset of unconjugated hyperbilirubinemia in the first week of life [9]. Although studies on jaundice due to bacterial infections have progressed, there are limited studies on jaundice caused by viral infections. Therefore, we investigated the relationship between asymptomatic RV infection and jaundice in neonates.

## Methods

### Study participants

A retrospective study of 1755 patients admitted to the neonatal intensive care unit (NICU) of Chonbuk National University Hospital through local obstetrics and gynecology hospitals and outpatient clinics was conducted from June 2014 to June 2017. Only patients aged 3–28 days at the time of admission were included in the study, to exclude patients within the incubation period of rotavirus and early jaundice due to blood type incompatibility. Patients were excluded from the study if they died within 3 days of admission or if there were no test results or records after admission. Finally, 596 patients were included in the analysis. During the study period, all admitted patients underwent RV screening tests and were screened daily for gastrointestinal symptoms. The demographic features that were evaluated included gestational age (GA), birth weight, small for gestational age, sex, age at admission, hospital day, mode of delivery, Apgar score (1 min/5 min), perinatal problems, and breastfeeding. Jaundice was diagnosed according to total bilirubin level and GA, in accordance with the American Academy of Pediatrics criteria [10]. Diarrhea was defined as the passage of unusually loose or watery stools, at least three times in a 24-h period (≥3 times/day or ≥ 2-fold increase in the number of stools per 8 h) [11]. Diarrhea was ascertained by the nurse at the time of admission, and by the nurse and doctor if the symptoms occurred after admission. Frequent passing of formed stools was not considered diarrhea. Babies fed only breast milk often pass loose, “pasty” stools; this was also not considered diarrhea. Vomiting was defined as the forceful expulsion of gastric contents occurring at least once in a 24-h period and without a respiratory or structural gastrointestinal tract cause [12]. Fever was defined as an axillary temperature of ≥38 °C. Perinatal problems included premature rupture of membranes, maternal diabetes mellitus, maternal thyroid disease, and maternal infection. Antibiotics were used during the period of hospitalization. Late-onset sepsis was considered when infection was detected in blood and cerebrospinal fluid cultures 7 days after delivery, caused by postnatal acquisition (with nosocomial or community sources) of the pathogen. In addition, respiratory viral polymerase chain reaction (PCR) was screened at the time of admission. Patients who tested positive for rotavirus were maintained on conservative therapy, such as fluid therapy and phototherapy until the stool test yielded negative findings in the isolation room.

### Clinical procedures

All patients underwent stool examination, respiratory viral PCR, and blood tests on admission. The stool test was performed within 24 h of admission. The RIDASCREEN® (R-Biopharm AG, An der Neuen Bergstraße 17, Germany) RV test is an enzyme immunoassay for the qualitative determination of RV in stool samples. The stool test was performed using enzyme-linked immunosorbent assay (ELISA), and the clinical records were retrospectively analyzed. The RIDASCREEN® Rotavirus was validated by comparison with three commercial rotavirus ELISAs. The sensitivity was 82.1% and specificity was 100% [13]. Blood tests included reticulocyte count, total bilirubin, direct bilirubin, hemoglobin (Hb) level, Coombs test, ABO incompatibility, C-reactive protein (CRP), and blood culture. The cut off value of total bilirubin was 15 mg/dL and that of CRP was 5 mg/L in this study [14, 15]. The respiratory viral PCR panel consists of multiple tests for respiratory virus influenza A/B, respiratory syncytial virus A/B, parainfluenza virus 1/2/3, coronavirus 229E/OC43/HKU1, rhinovirus A/B, adenovirus, human metapneumovirus, parainfluenza virus 4, bocavirus 1/2/3/4, enterovirus, coronavirus NL63, and Flu A-H1/H3/H1N1.

### Statistical analysis

Continuous variables are expressed as mean ± standard deviation, while categorical variables are expressed as number and percentage. Comparisons between continuous variables were performed using the Mann–Whitney *U* test or Student’s *t*-test, while comparisons between categorical variables were performed using the chi-squared test or Fisher’s exact test. Logistic regression analysis was performed to control for all variables and to determine the significant independent factors associated with jaundice. Adjusted odds ratios (ORs) and 95% confidence intervals (CIs) were calculated for each possible associated factor. Nominal values, such as sex, mode of delivery, presence or absence of RV infection, ABO incompatibility, blood culture, perinatal problems, and breastfeeding pattern, were compared using the χ^2^ test. Continuous variable parameters, such as GA, birth weight, age on admission, levels of serum total bilirubin, Hb level, reticulocyte count, Apgar score, CRP, and antibiotic use days were compared using an independent sample *t*-test. Statistical analyses were performed using SPSS version 18.0 (SPSS Inc., Chicago, IL, USA) and statistical significance was set at a *p* value of < 0.05.

## Results

A total of 596 patients were included in the study. Among these, 90% of infants were born at term (37–42 weeks of gestation) and 357 (60%) were born via vaginal delivery. The mean age of the study population was 11.4 ± 0.3 days. The mean birth weight was 3230.6 ± 508.3 g, and 338 (56%) infants were male and 258 (44%) were female. Among all patients, 166 (27.9%) were diagnosed with jaundice. The number of patients diagnosed with RV infection was 177 (29.7%). There was no significant seasonal variation in this study period.

Table 1 presents the demographic characteristics of patients with and without jaundice. There was no significant difference between the two groups for GA, birth weight, small for gestational age (SGA), sex, Apgar score, and perinatal problems. Perinatal problems included two cases of maternal diabetes mellitus, two cases of maternal thyroid disease, and 32 cases of premature rupture of membranes (PROM). At the time of admission, the mean ages of the jaundice and non-jaundice groups were 8.3 ± 0.4 days and 12.6 ± 0.4 days, respectively (*p* = 0.001). Hospital stay was shorter in the jaundice group than in the non-jaundice group (5.1 ± 0.6 days vs. 7.1 ± 0.4 days, respectively, *p* = 0.007. The rate of vaginal delivery in the jaundice group was significantly higher than that in the non-jaundice group [117 (70.5%) vs. 240 (55.8%), respectively, *p* = 0.001]. The rate of exclusive breastfeeding was significantly higher in the jaundice group than in the non-jaundice group [29.5% (49/166) vs. 16.5% (71/430), respectively, *p* = 0.001].

**Table 1:** Demographic characteristics of patients with and without jaundice

Characteristics	Jaundice (+) (*n* = 166)	Jaundice (−) (*n* = 430)	*p* value
Gestational age (weeks)	38.5 ± 0.1	39.0 ± 0.1	0.140
< 37 weeks (%)	8 (4.8)	53 (12.3)	
Birth weight (g)	3287.4 ± 38.4	3208.7 ± 24.7	0.090
Small for gestational age, n (%)	2 (1.2)	9 (2.1)	0.473
Male (%)	73 (44.0)	184 (42.8)	0.817
Age at admission (days)	8.3 ± 0.4	12.6 ± 0.4	0.001
Hospital days (days)	5.1 ± 0.6	7.1 ± 0.4	0.007
Vaginal delivery, n (%)	117 (70.5)	240 (55.8)	0.001
Apgar score (1 min)	8.8 ± 0.1	8.8 ± 0.1	0.741
Apgar score (5 min)	9.6 ± 0.2	9.8 ± 0.1	0.417
Perinatal problem, n (%)	14 (8.4)	22 (5.1)	0.131
Exclusive breastfeeding, n (%)	49 (29.5)	71 (16.5)	0.001

Table 2 presents a comparison of the factors that may affect jaundice. Reticulocyte counts demonstrated a significant difference of 2.3% ± 0.2% in the jaundice group and 1.9% ± 0.1% in the non-jaundice group (*p* = 0.029). Total bilirubin, direct bilirubin, and Hb levels were significantly higher in the jaundice group than in the non-jaundice group (*p* = 0.001). CRP level was significantly different in the jaundice group at 2.1 ± 0.4 mg/L and non-jaundice group at 7.2 ± 0.9 mg/L (*p* = 0.001). In the jaundice group, the RV infection rate was 70 (42%) and that of asymptomatic RV infection was 36 (21%), which was significantly different from the non-jaundice group (*p* = 0.001). There was no statistically significant difference in late-onset sepsis between the two groups. Respiratory PCR demonstrated a significant difference of 6 (3.6%) in the jaundice group and 61 (14.2%) in the non-jaundice group (*p* = 0.001). Among the 166 patients with jaundice, 63 (37.9%) used antibiotics.

**Table 2:** Associated factors of patients with and without jaundice

Characteristics	Jaundice (+) (*n* = 166)	Jaundice (−) (*n* = 430)	*p* value
Reticulocyte count (%)	2.3 ± 0.2	1.9 ± 0.1	0.029
Total bilirubin level (mg/dL)	18.7 ± 0.4	7.0 ± 0.2	0.001
Direct bilirubin level (mg/dL)	1.3 ± 0.1	0.8 ± 0.01	0.001
Hemoglobin level (mg/dL)	16.3 ± 0.2	14.6 ± 0.1	0.001
Coombs test positive, n (%)	5 (3.0)	1 (0.2)	0.560
ABO incompatibility, n (%)	2 (1.2)	1 (0.2)	0.133
C-reactive protein (mg/L)	2.1 ± 0.4	7.2 ± 0.9	0.001
Rotavirus positive, n (%)	70 (42.2)	107 (24.9)	0.001
Asymptomatic rotavirus positive, n (%)	36 (21.7)	31 (7.2)	0.032
Antibiotic use (days)	2.1 ± 3.5	3.0 ± 4.3	0.015
Late-onset sepsis, n (%)	1 (0.6)	4 (0.9)	0.695
Respiratory viral PCR, n (%)	6 (3.6)	61 (14.2)	0.001

*PCR* polymerase chain reaction

Table 3 present the jaundice and bilirubin levels in neonates with positive and negative RV infection. There was no difference in direct bilirubinemia with the presence of RV infection in the jaundice group. The total bilirubin level at ≤15 mg/dL was higher in the rotavirus positive group than in the rotavirus negative group (27.1% vs. 13.5%, respectively; *p* = 0.028).

**Table 3:** Jaundice and bilirubin levels in neonates with positive and negative RV infection

Characteristics	Rotavirus (+) (*n* = 70)	Rotavirus (−) (*n* = 96)	*p* value
Direct bilirubinemia (≥2 mg/dL)	3 (4.3)	9 (9.4)	0.211
Jaundice diagnosed			0.001
3–6 days old, n (%)	51 (72.9)	39 (40.6)	
≥7 days old, n (%)	19 (27.1)	57 (59.4)	
Total bilirubin level			0.028
≤15 mg/dL, n (%)	19 (27.1)	13 (13.5)	
> 15 mg/dL, n (%)	51 (72.9)	83 (86.5)	
C-reactive protein (mg/L)	1.9 ± 0.4	2.2 ± 0.5	0.604

Table 4 presents the results of the logistic regression analysis performed with all factors, seven of which demonstrated statistically significant differences. The risk of jaundice increased when the patient was older than 37 weeks, when the mode of delivery was vaginal, when the newborn was exclusively breastfed, when the Hb level was ≥15 mg/dL, and when tests for RV infection were positive (all *p* < 0.05). The risk of jaundice decreased when the age at admission was ≥7 days compared to that when age at admission was < 7 days [OR = 0.29; 95% CI, 0.16–0.51; *p* = 0.001]. The risk of jaundice decreased when the CRP level was ≥5 mg/dL compared to that when the CRP level was < 5 mg/dL [OR = 0.34; 95% CI, 0.20–0.61; *p* = 0.001].

**Table 4:** Multivariate analysis of the risk factors for jaundice

Association factors		Univariate analysis		Multivariate analysis	
		Odds ratio	*P* value	Odds ratio	*P* value
Gestational age	≥37 weeks	2.78 (1.29–5.97)	0.009	2.82 (1.23–6.49)	0.015
	< 37 weeks	1.00 (reference)		1.00 (reference)	
Birth weight	≥3.2 kg	1.06 (0.74–1.52)	0.746		
	< 3.2 kg	1.00 (reference)			
Sex	Male	0.96 (0.67–1.38)	0.833		
	Female	1.00 (reference)			
Age at admission	≥7 days	0.35 (0.24–0.51)	0.001	0.29 (0.16–0.51)	0.001
	< 7 days	1.00 (reference)		1.00 (reference)	
Vaginal delivery	Yes	1.89 (1.29–2.78)	0.001	1.83 (1.20–2.8)	0.005
	No	1.00 (reference)		1.00 (reference)	
Perinatal problem	Yes	1.37 (0.77–2.46)	0.288		
	No	1.00 (reference)			
Exclusive breastfeeding	Yes	2.12 (1.39–3.22)	0.001	3.91 (2.36–6.49)	0.001
	No	1.00 (reference)		1.00 (reference)	
Reticulocyte count	≥2%	1.51 (1.04–2.19)	0.029		
	< 2%	1.00 (reference)			
Hemoglobin level	≥15 mg/dL	3.56 (2.39–5.30)	0.001	3.05 (1.96–4.74)	0.001
	< 15 mg/dL	1.00 (reference)		1.00 (reference)	
C-reactive protein	≥5 mg/L	0.39 (0.23–0.65)	0.001	0.34 (0.20–0.61)	0.001
	< 5 mg/L	1.00 (reference)		1.00 (reference)	
Rotavirus test	Positive	2.20 (1.51–3.21)	0.001	1.89 (1.20–2.98)	0.006
	Negative	1.00 (reference)		1.00 (reference)	
Late-onset sepsis	Yes	0.65 (0.07–5.82)	0.645		
	No	1.00 (reference)			

## Discussion

An important finding of our study is that RV infection was associated with neonatal jaundice. The prevalence of jaundice was 27.8% among all patients admitted to NICU, and among jaundiced patients, 42.2% had positive tests for RV infection. In addition, 22% of jaundiced patients demonstrated asymptomatic RV infection. The incidence of jaundice was higher in the RV-positive group.

RV is now known to be an important causative agent of hospital infections. An electrophoretic analysis revealed that 41% of RV infections occur during winter and 30% of asymptomatic infections occur in other seasons [16]. In addition, other reports on RV infection in neonates suggested a high infection rate with few signs or symptoms [17]. Only 14 (23%) infants infected with RV experienced fever, vomiting, and diarrhea [12]. Several factors have been proposed to be responsible for asymptomatic RV infection observed in neonates, including host features such as physiological immaturity of the neonatal gut, the role of maternal antibodies, and virulence characteristics of the unique neonatal strains. It is known that asymptomatic infants usually develop asymptomatic infections due to the newborn strain, which is completely different from the RV strain found in pediatric patients, but it is known to cause diarrhea, dehydration, jaundice, metabolic acidosis, necrotizing enteritis, and even death [18]. Identifying neonates who are infected with RV is important for infection control in the NICU. The prevalence of RV in hospitals is high, but once mass incidence occurs, it is difficult to block transmission, despite proper patient isolation [19]. Moreover, most infected neonates have no specific enteritis symptoms and go through an incubation period. Therefore, infants with asymptomatic jaundice caused by RV infection should be screened for rapid isolation.

Jaundice is common and is associated with a variety of physiological and pathological conditions. It is the main reason for hospital readmission during the neonatal period and has long been recognized as a clinical manifestation of infection in the neonate and early infancy [7]. It is known that various infections such as neonatal sepsis, congenital infection, and hepatitis are risk factors for neonatal jaundice. Previous studies have demonstrated that urinary tract infections were associated with jaundice, but there is no such study on RV [8, 9, 20, 21]. In our study of the jaundice group, total bilirubin levels were less than 15 mg/dL in the rotavirus infection group. Rotavirus-induced jaundice did not increase the total bilirubin level more than in other causes. In addition, rotavirus infection is diagnosed early in the disease because it can be screened before other causes.

Besides RV infection, neonates born at term, born via vaginal delivery, those who are exclusively breastfed, and who have high Hb were at high risk in this study. Breastfeeding was associated with neonatal jaundice that lasted longer and reached higher peak levels than milk feeding [21]. Jaundice in breastfed babies usually appears between 24 and 72 h after birth, peaks by 5–15 days of life, and disappears by the third week of life; high bilirubin levels have been reported in such infants [22]. While exclusive breastfeeding has historically been an important predictor for jaundice, the mechanism underlying the association is not well understood. Our results demonstrate that vaginal delivery was associated with jaundice. The present findings indicate that naturally delivered neonates were more likely to have jaundice than those born via cesarean section. Previous studies have shown that the risk of jaundice increases in children delivered via cesarean section [5]. Similar to our study, Bulbul et al. [23] reported a statistically significant correlation between the severity of jaundice and mode of delivery. However, other studies have yielded controversial results regarding the relationship between labor and jaundice. Some studies demonstrated a lack of significant relationship between mode of delivery and jaundice [24].

Our study has several limitations owing to its retrospective nature. It did not include healthy infants and was targeted toward infants admitted to the NICU. Although the RIDASCREEN® RV test has high sensitivity to RV infection, there is a possibility of false positives in the RV test results. Therefore, asymptomatic RV-positive patients can have false-positive results. In addition to the exclusion of 1159 (66%) patients, we excluded patients in the incubation period of rotavirus; this difference may lead to selection bias of the included patients or incorrect risk estimates. However, our study was also meaningful in that all patients admitted to the NICU were screened for RV infection.

## Conclusions

RV infection can occur in asymptomatic neonates presenting with jaundice. Jaundice may be the first sign of RV infection before other signs become evident. Thus, we suggest that RV screening tests be considered as a part of the diagnostic evaluation of neonates older than 3 days presenting with hyperbilirubinemia.

## Additional file

Additional file 1: The datasets of this study. Brief description of the data: The datasets supporting the conclusion of this study. (XLSX 157 kb)

## Abbreviations

CRP: C-reactive protein
GA: Gestational age
Hb: Hemoglobin
NICU: Neonatal intensive care unit
PCR: Polymerase chain reaction
PROM: Premature rupture of membrane
RV: Rotavirus
SGA: Small for gestational age
